# Army Dentistry

**Published:** 1919

**Authors:** 


**Army Dentistry.** By Lieut. A. R. LIVERMORE. *Public Health* (pub. by Michigan State Board of Health), October 1918.

There is less extracting per patient done than in civilian life. Crown, bridge and plate work are not done by the army dental surgeon; more effort is made to save teeth by proper insertion of amalgam fillings. Other fillings may be used, but the amalgam is the one most used by the army dentist. He also gives teeth necessary treatment, and treats the diseases of the oral cavity. In the treatment of pyorrhea, which is quite common, the army dentist has the advantage of having the patient under orders to report for treatment as often as necessary.

The army dentist also gives instruction to the men in oral hygiene, and makes a bi-monthly inspection.

